# Jiangtang decoction ameliorate diabetic nephropathy through the regulation of PI3K/Akt-mediated NF-κB pathways in KK-Ay mice

**DOI:** 10.1186/s13020-017-0134-0

**Published:** 2017-05-19

**Authors:** Jin-Ni Hong, Wei-Wei Li, Lin-Lin Wang, Hao Guo, Yong Jiang, Yun-Jia Gao, Peng-Fei Tu, Xue-Mei Wang

**Affiliations:** 1 Integrated Laboratory of Traditional Chinese Medicine and Western Medicine, Peking University First Hospital, Beijing, People’s Republic of China; 2 Institute of Basic Medical Sciences, Xiyuan Hospital, China Academy of Chinese Medical Sciences, Beijing, People’s Republic of China; 3 School of Pharmaceutical Science, Peking University, Beijing, People’s Republic of China

**Keywords:** Diabetic nephropathy, Jiangtang decoction, Inflammation, Traditional Chinese Medicine

## Abstract

**Background:**

Jiangtang decoction (JTD) is a China patented drug which contains *Euphorbia humifusa* Willd, *Salvia miltiorrhiza* Bunge, *Astragalus mongholicus* Bunge, *Anemarrhena asphodeloides* Bunge, and *Coptis chinensis* Franch. For decades, it has also been used clinically to treat diabetic nephropathy (DN) effectively; however, the associated mechanisms remain unknown. Thus, the present study aimed to examine the protective efficacy of JTD in DN and elucidate the underlying molecular mechanisms.

**Methods:**

A diabetic model using KK-Ay mice received a daily administration of JTD for 12 weeks. Body weight, blood glucose, triglycerides (TGs), total cholesterol (TC), urea nitrogen (UN), creatinine (Cr), and microalbumin/urine creatinine (MA/UCREA) was measured every 4 weeks. Furthermore, on the day of the sacrifice, blood, urine, and kidneys were collected to assess renal function according to general parameters. Pathological staining was performed to evaluate the protective renal effect of JTD. In addition, the levels of inflammatory cytokines (tumor necrosis factor-α [TNF-α], interleukin [IL]-6 and intercellular adhesion molecule [ICAM]-1), insulin receptor substrate [IRS]-1, advanced glycation end products [AGEs], and receptor of glycation end products [RAGE] were assessed. Finally, the phosphoinositide 3-kinase (PI3K)/protein kinase B (Akt) signaling pathway and involvement of nuclear factor-κB (NF-κB) was further analyzed.

**Results:**

After 12 weeks of metformin and JTD administration, the mice exhibited a significant amelioration in glucose and lipid metabolism dysfunction, reduced morphological changes in the renal tissue, decreased urinary albumin excretion, and normalized creatinine clearance. JTD treatment also reduced the accumulation of AGEs and RAGE, up-regulated IRS-1, and increased the phosphorylation of both PI3K (p85) and Akt, indicating that the activation of the PI3K/Akt signaling pathway was involved. Additionally, JTD administration reduced the elevated levels of renal inflammatory mediators and decreased the phosphorylation of NF-κB p65.

**Conclusions:**

These results demonstrate that JTD might reduce inflammation in DN through the PI3K/Akt and NF-κB signaling pathways.

**Electronic supplementary material:**

The online version of this article (doi:10.1186/s13020-017-0134-0) contains supplementary material, which is available to authorized users.

## Background

Diabetes mellitus (DM) is an endocrine-based metabolic disease characterized by hyperglycemia due to insufficient insulin or/and insulin resistance (IR) [1]. Glucose glycates proteins and generates advanced glycation end products (AGEs), which can induce cellular effects via interactions with specific cellular receptors. Receptor of glycation end products (RAGE) is an important signal transduction receptor that activates an array of signaling transduction cascades in response to AGE-binding [2]. Moreover, elevated levels of RAGE were found to activate pro-inflammatory nuclear factor-κB (NF-κB) and other inflammatory responses, so as to induce diabetic complications [3]. DN is one of these complications, for which early progression is notoriously difficult to detect and quantify prior to the occurrence of substantial histological damage, manifesting as microalbumin (MA) and declining glomerular filtration rate. Diabetic nephropathy (DN) is considered to be an inflammatory disease, and increased inflammatory factors contribute significantly to the development of DN [3, 4]. Moreover, NF-κB is a critical signaling pathway which mediates several inflammatory processes [5]. As an upstream mediator of NF-κB, the phosphoinositide 3-kinase (PI3K)/protein kinase B (Akt) signaling pathway has been confirmed to play a vital role in proliferation, cell cycle progression, and cell viability in DN [6]. In addition, rats with streptozotocin (STZ)-induced DN exhibit a significant decrease in the PI3K and Akt [7]. Moreover, podocyte apoptosis can be inhibited by stabilizing the PI3K/Akt signaling pathway [8]. These findings indicate that the PI3K/Akt signaling pathway might play a role in DN, and alternative approaches which activate the PI3K/Akt signaling pathway and inhibit NF-κB-dependent inflammation might be a potential method of protecting against renal injury.

In China, the incidence of DN ranges from 25 to 40% [9]. In addition, DN is both the strongest predictor of mortality in diabetic patients worldwide and the leading cause of end-stage renal disease, with limited effective therapies available. Therefore, further study of the mechanisms of DN, as well as prevention and treatment strategies are required. Based on the associated clinical manifestations, DM has been defined as “Xiao-Ke,” a sign of a Yin-Yang imbalance, characterized by yin vacuity with fire flaming upward, as well as qi vacuity [10]. Physically, “Xiao-Ke” patients experience dryness, thirst, and weight loss due to exhaustion. Based on the etiology and pathogenesis of DM, scholars in Traditional Chinese Medicine (TCM) have reported the main cause of DM to be heat toxins [10]. Specifically, the internal blazing of heat and stagnation of qi damage the yin, making it deficient [10]. In addition to “excessive fire consuming qi,” damage to yin and qi via internal heat has also been proposed as the primary mechanism of DM [10]. Thus, the effects of heat toxins on qi, yin, and blood stasis are thought to be key pathogenic factors in DM [10]. Other studies of TCM also indicate that diabetes may be an inflammatory disease and the inhibition of inflammation might be useful to prevent its development [11]. Moreover, previous studies found that Chinese herbal medicine, such as berberine [11], astragaloside IV [11], salvianolic acid B [12], and timosaponin B-II [13], one of the main components of *Coptis chinensis Franch*, *Astragalus mongholicus* *Bunge, Salvia* *miltiorrhiza* *Bunge,* and *Anemarrhena asphodeloides* *Bunge*, respectively, might exert hypoglycemic effects that are partially mediated by anti-inflammatory mechanisms. JTD, a Chinese patented drug (Patent Number: 20141002188.3) containing *Euphorbia humifusa* Willd, *S. miltiorrhiza* Bunge, *A. mongholicus* Bunge, *A. asphodeloides* Bunge, and *C. chinensis* Franch was specifically formulated to clear heat, promote blood circulation, supplement the qi, and nourish the Yin, has been used clinically for decades to effectively treat DN; however, the mechanisms remain unclear. Therefore, the aim of the present study was to examine the hypothesis that JTD ameliorates DN by inhibiting inflammation through the PI3K/Akt and NF-κB signaling pathways.

## Methods

The Minimum Standards of Reporting Checklist contains details of the experimental design, and statistics, and resources used in this study (Additional file 1).

### Preparation and quality control of JTD

*Euphorbia humifusa* Willd, *S. miltiorrhiza* Bunge, *A. mongholicus* Bunge, *A. asphodeloides* Bunge, and *C. chinensis* Franch were purchased at the Chinese Medicine Pharmacy at Peking University First Hospital (No. 8, Xishiku Street, Xicheng District, Beijing, PRC). The medicinal components of JTD are listed in Table 1. Ethanol extracts were prepared in six volumes of 60% alcohol, then soaked for 30 min, and then refluxed twice for 1 h. The herbs were then filtered, condensed, and dried. Metformin HCl (1 g) was dissolved in 40 mL ddH_2_O and mixed. The components of JTD were detected using high-performance liquid chromatography (HPLC; Agilent 1100, USA).

**Table 1:** Composition of JTD

Species	Chinese name	Plant part	Family	Mass (g)
*Euphorbia humifusa* Willd	Dijincao	Leaf	Euphorbiaceae	15
*Salvia miltiorrhiza* Bunge	Danshen	Root	Lamiaceae	20
*Astragalus mongholicus* Bunge	Huagnqi	Root	Leguminosae	10
*Anemarrhena asphodeloides* Bunge	Zhimu	Root	Asparagaceae	10
*Coptis chinensis* Franch	Huanglian	Root	Ranunculaceae	5
Total amount				60

Appropriate amounts of JTD ethanol extract were dissolved and diluted with methanol to a concentration of 25 mg/mL. The sample solutions were then filtered through a 0.22-μm polytetrafluoroethylene membrane prior to performing HPLC analysis. Chromatographic separation was carried out in a Gemini-NX C18 column (250 mm × 4.6 mm, 5.0 μm, Phenomenex Inc.) at 30 °C with an acetonitrile (solvent A) and 15% acetonitrile-0.01% phosphoric acid aqueous solution (solvent B; 0.15 g SDS and 0.68 g monosodium phosphate were added to 500 mL of solvent B) as the mobile phases. The chromatographic conditions were optimized to acquire good separation within 60 min at a flow rate of 1.0 mL/min. The gradient program was set as follows: 0–5 min, 0% A; 5–20 min, 0–25% A; 20–30 min, 25–35% A; 30–40 min, 35–50% A; 40–50 min, 50–60% A; and 50–60 min, 60–80% A. The wavelength used for detection was 250 nm.

### Animal grouping and drug administration

All animal experiments were performed with the approval of the Institutional Animal Care and Use Committee of Peking University First Hospital (Approval Number: J201534). Male KK-Ay mice (*N* = 50) and C57BL/6J mice (*N* = 10) were purchased from Beijing HFK Bioscience Co., Ltd. [License No. SCXK (Jing) 2012-0001]. The mice were aged 8–9 weeks old and weighed 30 ± 2 g. Blood glucose was measured via the glucose oxidase method. All KK-Ay mice with PPG ≥11.1 mmol/L or FG ≥7.0 mmol/L and MA tested in the urine were used as DN samples, and all others were excluded. The mice recruited were randomly divided into six groups as follows: (1) control (untreated C57BL/6J mice); (2) model (untreated KK-Ay mice); (3) metformin (KK-Ay mice treated with 250 mg/kg metformin); (4) low JTD (KK-Ay mice treated with 2 g/kg ethanol extract of JTD); (5) medium JTD (KK-Ay mice treated with 4 g/kg ethanol extract of JTD); and (6) high JTD (KK-Ay mice treated with 8 g/kg JTD ethanol extract). All drugs were administered orally once per day for 12 weeks.

### Metabolic parameters and renal function analysis

After 4, 8 and 12 weeks of treatment, the mice were placed in metabolic cages for 24-h urine collection, food, and water intake calculations. The blood was collected from the mouse caudal veins. Blood glucose, triglycerides (TGs), total cholesterol (TC), urea nitrogen (UN), creatinine (Cr), and microalbumin/urine creatinine (MA/UCREA) in the urine and/or serum were measured using a Hitachi 7180 biochemical analyzer (Hitachi Ltd., Tokyo, Japan). The kidneys were removed and frozen at −80 °C until processed for Western blotting and RNA extraction. The remaining kidney was used for analysis by light and electronic microscopy.

### Histopathological analysis

After 12 weeks of treatment, the kidneys were removed and fixed in 10% formaldehyde and embedded in paraffin. Sections (4-μm thick) were cut and stained with hematoxylin and eosin (H&E) and periodic schiff-methenamine (PASM) modified trichrome histological stains. The stained sections were examined at 400× magnification by an observer blinded to the treatment group of the tissue slices. In each section, 20 glomeruli were randomly selected, and the ratio of the mesangial matrix area to the total glomerular area (M/G) in the PASM-stained sections was determined using Image-Pro Plus quantitative software (Pax-it; Paxcam, Villa Park, IL, USA).

### Immunohistochemistry analysis

The kidneys were immersed in 4% paraformaldehyde for 4 h and then transferred to 70% ethanol. Then the samples were cut into 4-μm-thick paraffin-embedded kidney sections and were incubated at 4 °C with AGEs (Abcam, UK) or RAGE (Abcam, UK) overnight. The sections were then incubated with a goat anti rabbit secondary antibody (Beijing Zhong Shan Golden Bridge Biotechnology Co., Ltd., China), for 1 h at room temperature. A negative control was included, in which the primary antibody was preincubated with a control peptide. Sections were examined using an Olympus DY07 microscope (Olympus, Tokyo, Japan) and digitized with a high-resolution camera (magnification 400×). Glomeruli or tubulointerstitial areas (*N* = 20, each) were randomly selected from each section and immunostaining was performed on the AGEs and RAGE. Immunostaining signals within each selected glomerular or tubulointerstitial area were highlighted and quantified using Image-Pro Plus quantitative software (Pax-it; Paxcam, Villa Park, IL, USA). Regions with positive staining were quantified and expressed as a percentage of the entire glomerulus.

### Ultrastructural analysis

After 12 weeks of treatment, the renal cortexes of the mice were removed and fixed in 3% glutaraldehyde at 4 °C and embedded in an epoxy resin. Ultrathin sections were double-stained with 1.25% uranium acetate and 0.4% lead citrate, and examined under a JEM-100C X II electron microscope (JEOL Ltd., Tokyo, Japan).

### ELISA analysis

After 12 weeks of treatment, the sera were collected. The concentrations of TNF-α (Nanjingjiancheng Inc., Nanjing, China), IL-6 (Nanjingjiancheng Inc., Nanjing, China), AGEs (Abbexa Ltd., UK), and RAGE (RnD bio-techne, USA) in the serum were measured using commercial ELISA kits according to the manufacturer’s protocol.

### Western blot analysis

After 12 weeks of treatment, the kidneys from each of the mice were collected and prepared using a protein extraction kit (KetGEN Biotech Inc., Nanjing, China) according to the manufacturer’s protocol. Equal quantities of protein were separated using sodium dodecyl sulfate (SDS)-polyacrylamide gel electrophoresis and transferred to a polyvinylidene fluoride membranes. The membranes were blocked with 5% bovine serum albumen (Amresco, Inc. USA) and incubated with the primary antibodies: AGEs (Abcam, UK), RAGE (Abcam, UK), PI3K p85 (cell signaling technology, USA), p-PI3K p85 (cell signaling technology, USA), Akt (pan) (cell signaling technology, USA), p-Akt (Ser473) (cell signaling technology, USA), IκBα (cell signaling technology, USA), NF-κB p65 (cell signaling technology, USA), p-NF-κB p65 (Santa Cruz Biotechnology, Glostrup, Denmark) and GAPDH (Beijing Zhong Shan Golden Bridge Biotechnology Co., Ltd., China), with a dilution of 1:1000, overnight at 4 °C. Following incubation with a horseradish peroxidase-labeled secondary antibody (Beijing Zhong Shan Golden Bridge Biotechnology Co., Ltd.,) at room temperature for 1 h, the membranes were developed with enhanced chemiluminescence (Thermo Scientific, USA) and visualized using a digital imaging system (BIO-RAD Laboratories, Inc., USA).

### Real-time PCR analysis

The total RNA was extracted from the kidneys using Trizol Reagent (Invitrogen, USA). The cDNA was synthesized using a High-Capacity cDNA Reverse Transcription Kit (Applied Biosystems, CA, USA). Quantitative real-time PCR was performed using the Power SYBR Green PCR Master MIX (Applied Biosystems, CA, USA) with the primers presented in Table 2 using the Fast Real-Time PCR System 7500 (Applied Biosystems, CA, USA). The relative level of mRNA expression was calculated using the 2^−ΔΔCt^ method. The expression of GAPDH mRNA was used as the endogenous reference control.

**Table 2:** Nucleotide sequences of the primers used for real-time PCR

	Forward nucleotide sequence 5′–3′	Reverse nucleotide sequence 5′–3′
*IRS*-*1*	GGCAGCAATGAGGGCAACT	TGGAAGCTGATGCTGGCATA
*IL*-*6*	GTTGCCTTCTTGGGACTGAT	GCCATTGCACAACTCTTTTCT
*TNF*-*α*	GCCATTGCACAACTCTTTTCT	AGTTGGTCCCCCTTCTCC
*ICAM*-*1*	GCTTCACACTTCACAGTTACTT	AGAGGACCTTAACAGTCTACAAC
*PI3K*	CGGCGTGACATGTAGGCTCTCA	ACGGCCCGCACTGTAACCTAT
*Akt*	TGTGACCATGAACGAGTTTGA	GTCGTGGGTCTGGAATGAGTA
*IKKβ*	TGCTGCCCAAGGTAGAAGAGGTAG	AGGCACTGGAAGGCTGGGACATTA
*NF*-*κB*	CACCTCACCGGCCTCATCCACA	AGTCCCCGCGCTGCTCCTCTAT
*GAPDH*	GGTGGAGCCAAAAGGGTCAT	CGTGGTTCACACCCATCACA

### Statistical methods

All data were analyzed with SPSS 19.0 statistical software, and the data are reported as the mean ± standard deviation (SD). The statistical analysis between the different groups was performed using a one-way analysis of variance (ANOVA) with a Dunnets’s multiple comparison post hoc test. *P* < 0.05 was considered to be statistically significant.

## Results

### Content of the JTD ethanol extract

**Fig. 1:**
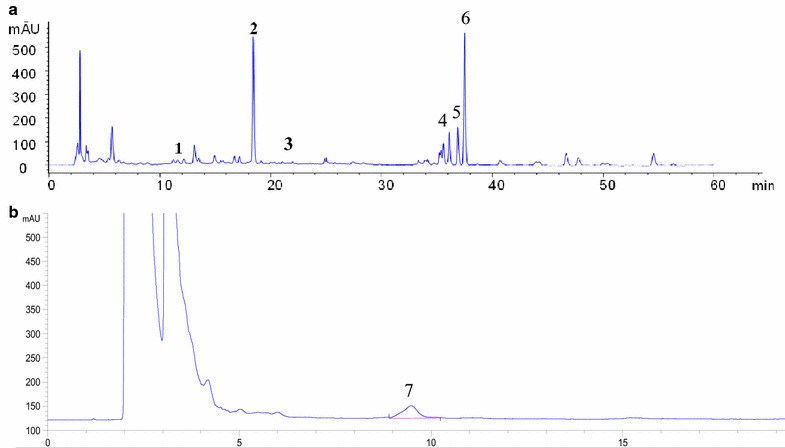
The HPLC of the FTD ethanol extract. **a** The *numbers* shown in the chromatograms indicate the peaks of calycosin-7-*O*-β-d-glucoside (*1*), salvianolic acid B (*2*), quercetin (*3*), coptisine (*4*), palmatine (*5*), berberine (*6*); **b** The numbers shown in the chromatograms indicate the peaks of timosaponin BII (*7*)

After repeating the HPLC examination for JTD, the fingerprint revealed that the following seven of the components listed below are the main constituents of JTD. The HPLC profile of JTD ethanol extract (Fig. 1) exhibits peaks corresponding to calycosin-7-*O*-β-d-glucoside, which is the main component of *A. mongholicus* Bunge; salvianolic acid B, the main component of *S. miltiorrhiza* *Bunge*; quercetin, the main component of *E. humifusa* Willd; coptisine, palmatine, and berberine, three main components of *C. chinensis* Franch; Timosaponin BII, the main component of *A. asphodeloides* Bunge. And the quantification of JTD are listed in Table 3.

**Table 3:** Quantification of JTD

Component	Ratio (mg/g JTD)
Calycosin-7-*O*-β-d-glucoside	0.15
Salvianolic acid B	1.89
Quercetin	0.05
Coptisine	14.12
Palmatine	0.61
Berberine	2.53
Timosaponin BII	0.68

### JTD reduces urinary albumin excretion, as well as ameliorates kidney function and metabolism

**Fig. 2:**
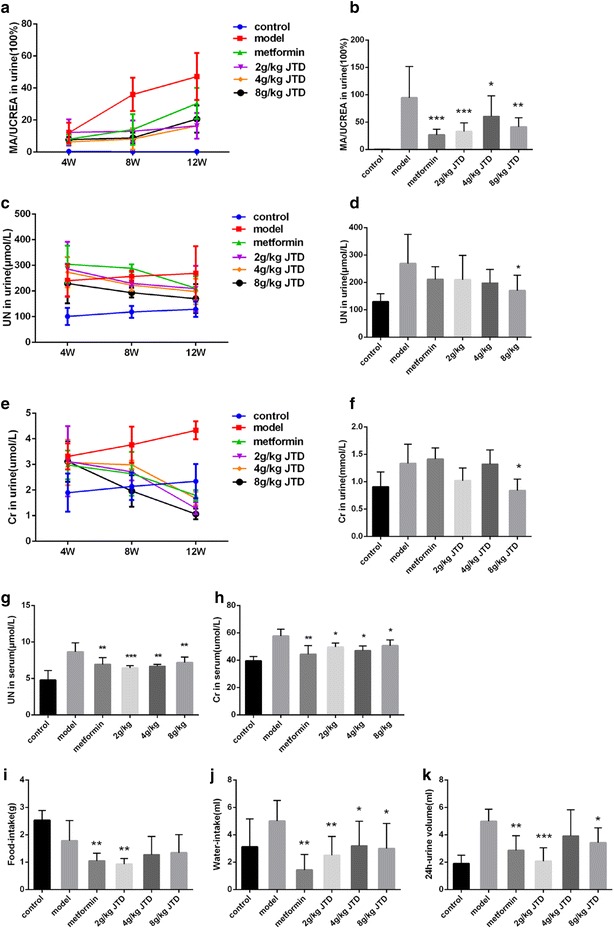
JTD reduces urinary albumin excretion, as well as ameliorates the kidney function and metabolism of KK-Ay mice. **a** Changes of MA/UCREA in the urine during the treatment period. **b** JTD reduces the level of MA/UCREA in the urine. **c** Changes in the level of UN in the urine during the treatment period. **d** JTD reduces the level of UN in the urine. **e** Changes of Cr in the urine during the treatment period; **f** JTD reduces Cr in the urine. **g** JTD reduces the level of UN in the serum. **h** JTD reduces Cr in the serum. **i** JTD reduces food intake. **j** JTD reduces water intake. **k** JTD reduces the 24-h urine volume. Data are expressed as the mean ± S.D.; *N* = 10; *P < 0.05; **P < 0.01; and ***P < 0.001 compared to the model group

As shown in Fig. 2a, MA/UCREA was notably elevated in the DN model group over time compared to the control group, while the combined metformin and JTD treatment were able to alleviate the increase of MA/UCREA in the urine. After 12 weeks of treatment with metformin and JTD, there was a significant decrease in MA/UCREA compared to the model group (Fig. 2b). Furthermore, treatment with JTD were found to down-regulate the concentration of UN and Cr in the urine (Fig. 2c, e) over time, thereby normalizing the concentration of UN in the urine in a dose-dependent manner (Fig. 2d) and decreasing the level of Cr excretion in the urine (Fig. 2f) after 12 weeks of treatment. The level of UN and Cr in the serum were tested after 12 weeks of treatment. Metformin and JTD treatment were found to significantly reduce UN and Cr in the serum (Fig. 2g, h). In addition, the mice were put into metabolic cages for 24 h, and the level of food intake, water intake, and urine volume were calculated. The results showed that after 12 weeks of treatment with JTD and metformin, there was decreased food intake, water intake, and urine volume (Fig. 2i–k). It is interesting to note that treatment with a low dose of JTD can significantly normalize metabolism when compared with the model group.

### JTD alleviates renal histopathology and ultrastructural pathology of the kidneys

**Fig. 3:**
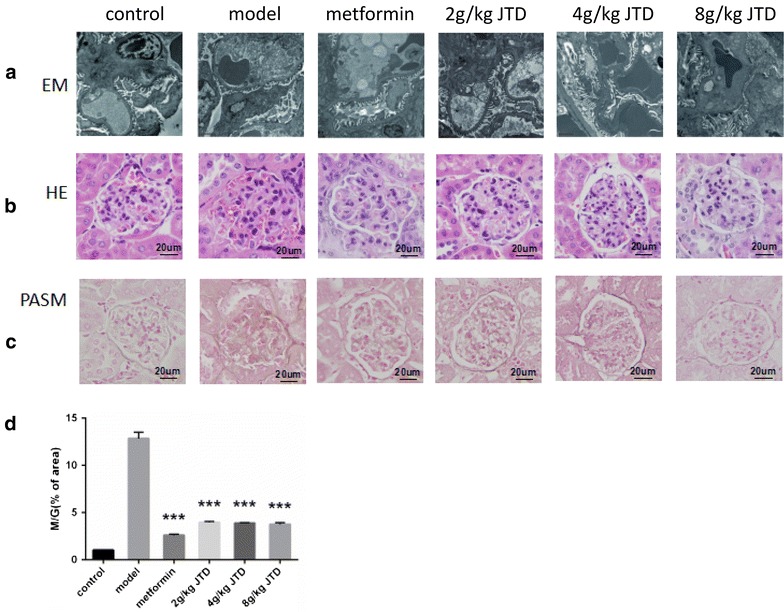
JTD alleviates renal histopathology and the ultrastructural pathology of the kidney. **a** Electron microscopy (EM) analysis of the renal cortex, representative images of glomerular basement membrane thickening and mesangial matrix expansion (*scale bar* 2 μm; original magnification electron microscopy ×8000; **b** hematoxylin and eosin (HE) staining of the kidney (original magnification ×400); **c** periodic schiff-methenamine (PASM) staining of the kidney (original magnification ×400); **d** JTD lowers the ratio of the mesangial matrix area to the total glomerular area (M/G) via PASM staining. Data are expressed as the mean ± S.D.; *N* = 5; *P < 0.05; **P < 0.01; and ***P < 0.001 compared to the model group

Electron microscopy of the renal cortex ultrastructure revealed mesangial expansion, mesangial matrix deposition, podocyte fusion, and glomerular basement membrane thickening in the model group; however, these changes were significantly alleviated in the JTD and metformin-treated groups (Fig. 3a).

Both H&E and PASM staining revealed that compared to the control group, KK-Ay in the model group exhibited more severe pathological changes. From H&E staining, we found that the glomerular capillaries were dilated and filled with large amounts of red blood cells, and plasma proteins had extravasated from the renal capsules (Fig. 3b). From the PASM staining, notable glomerular hypertrophy, basement membrane thickening, increased mesangial matrix area, and glycogen, as well as vacuolation and deformation of the renal tubules were observed in the model group. Moreover, treatment with JTD or metformin significantly ameliorated these changes (Fig. 3c). Additionally, PASM staining showed that the M/G ratios were higher in the model group than the controls, whereas treatment with JTD and metformin significantly lowered the M/G ratio (Fig. 3d).

### JTD reduce the increase in blood glucose, weight, and lipid metabolism

**Fig. 4:**
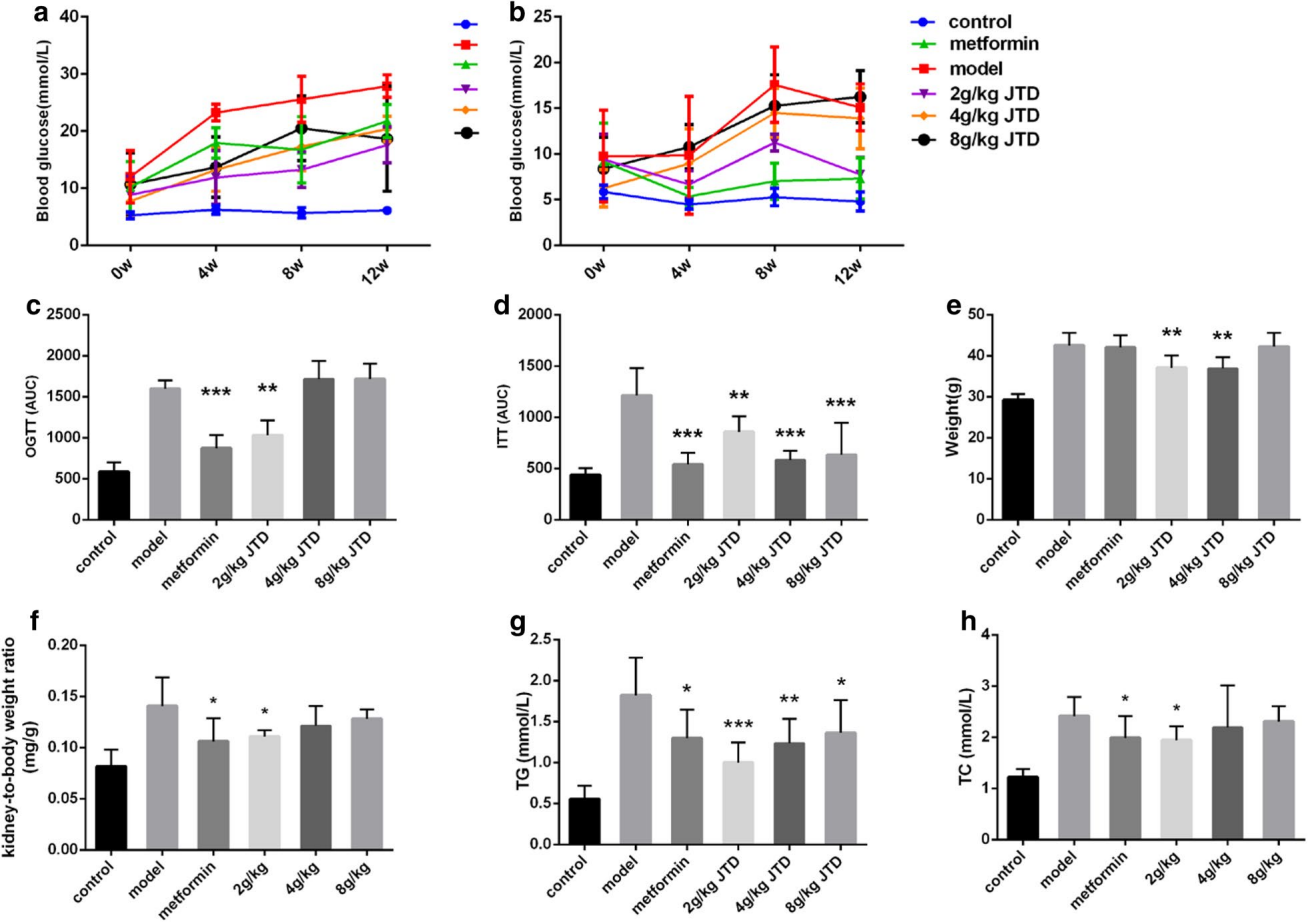
JTD reduces elevated levels of blood glucose, weight, and lipid metabolism. **a** JTD reduces PPG; **b** JTD reduces FG; **c** JTD reduces the area under the curve of OGTT; **d** JTD reduces the area under the curve of ITT; **e** JTD reduces the weight of mice; **f** JTD reduces the kidney to body weight ratio; **g** JTD reduces TGs; **h** JTD reduces TC. Data are expressed as the mean ± S.D.; *N* = 10; *P < 0.05; **P < 0.01; and ***P < 0.001 compared to the model group

DM is a metabolic disease characterized by a high concentration of blood glucose. Thus, we examined the glucose levels of mice every 4 weeks and found that treatment with an oral administration of metformin and JTD decreased the increased levels of glucose (Fig. 4a, b). As shown in Fig. 4a, a significant increase in postprandial glucose (PPG) in KK-Ay mice was decreased by metformin and JTD. Additionally, the effect of a low and medium dose of JTD was more potent than that of metformin. As shown in Fig. 4b, JTD can also reduce elevated levels of fasting glucose (FG), with a low and medium dose of JTD demonstrating a greater effect than a high dose of JTD. Moreover, JTD can reduce the area under the curve (AUC) of both the oral glucose tolerance test (OGTT) and insulin tolerance test (ITT) in KK-Ay mice (Fig. 4c, d). From Fig. 4c, it is observed that metformin and low doses of JTD can reduce OGTT (AUC), whereas a medium and a high dose of JTD have no positive effect. In Fig. 4d, both metformin and JTD are shown to significantly down-regulate ITT (AUC), compared to the model group. In addition, after 12 weeks of administration, body weight was found to be markedly increased in the model mice compared to the other groups (Fig. 4e). Furthermore, metformin and JTD were found to down-regulate the kidney to body ratio after 12 weeks of JTD administration (Fig. 4f). Additionally, we found that treatment with JTD significantly lowered the level of TGs and TC, which were elevated in the model group in a dose-dependent manner (Fig. 4g, h).

### JTD reduces the accumulation of AGEs and RAGE

**Fig. 5:**
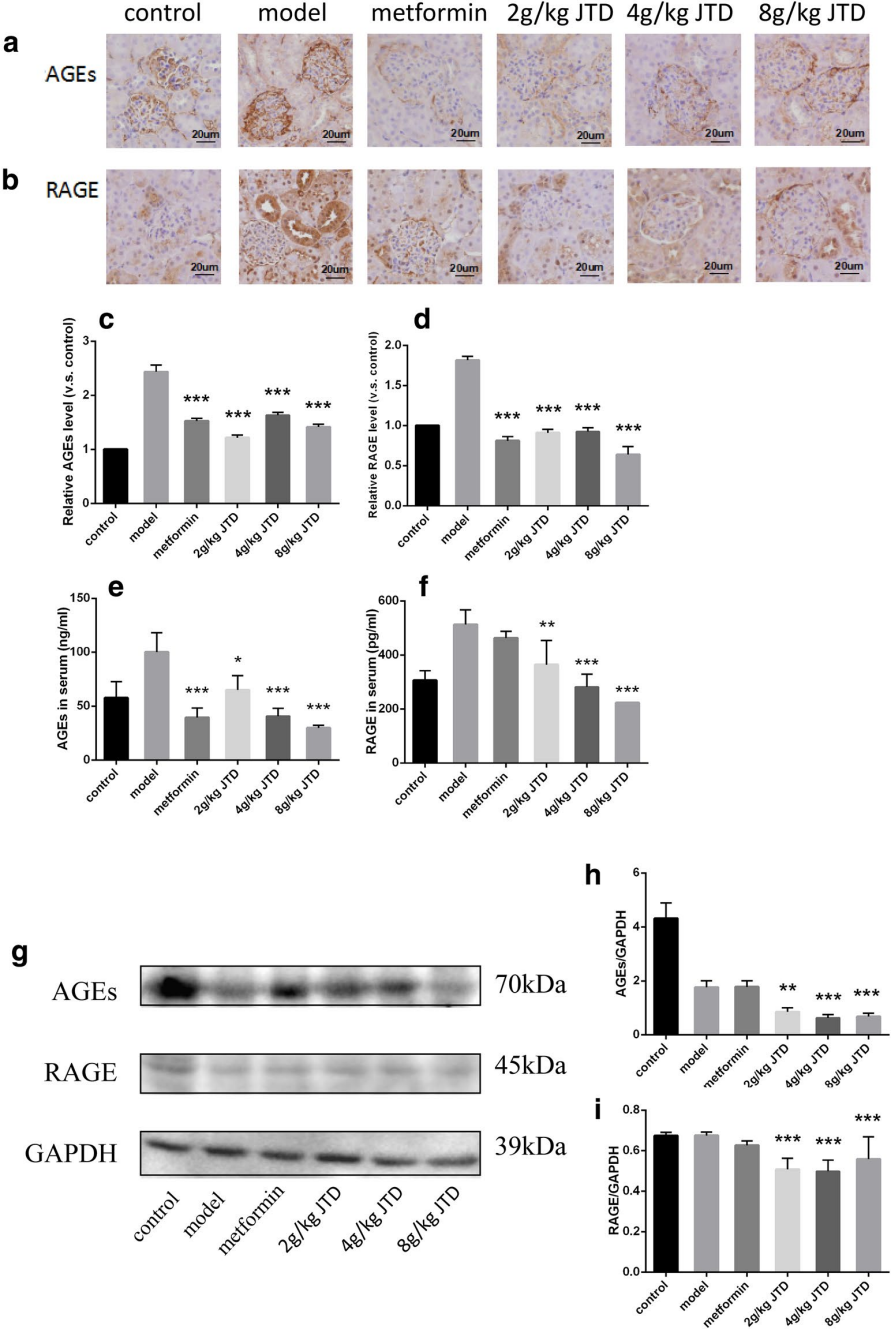
JTD reduces the accumulation of AGEs and RAGE. **a** Immunohistochemistry of AGEs (original magnification ×400); **b** immunohistochemistry of RAGE (original magnification ×400); **c** JTD reduces the ratio of the area with AGEs accumulation to the total glomerular area; **d** JTD reduces the ratio of the area with RAGE accumulation to the total glomerular area; **g** Western blot analysis of AGEs and RAGE; **h** JTD can down-regulate the expression of AGEs; **i** JTD can down-regulate the expression of RAGE. Data are expressed as the mean ± S.D.; *N* = 10; *P < 0.05; **P < 0.01; and ***P < 0.001 compared to the model group

Non-enzymatic glycation of proteins by reducing saccharides is a post-translational modification leading to the formation of AGEs, which accumulate during aging and are involved in the pathogenesis of many diseases (e.g., DM) [14]. Studies on the pathological mechanisms of glycation-related diseases (e.g., DM) have shown that AGEs can induce cellular effects through interacting with specific cellular receptors [14]. In addition, RAGE is an important signal transduction receptor which activates an array of signal transduction cascades in response to AGE-binding [2]. Furthermore, both AGEs and RAGE have been identified as key regulators of DN [2]. Thus, we next examined whether AGEs and RAGE accumulate in diabetic kidneys and whether JTD can alleviate this accretion. As presented in Fig. 5a, b, immunohistochemistry staining of AGE and RAGE in renal tissue were performed to assess the regional changes of AGEs and RAGE accumulation in diabetic kidneys. It was apparent that treatment with JTD and metformin could reduce the ratio of the area of AGE and RAGE accumulation to the total glomerular area (Fig. 5c, d). To elucidate the detailed mechanisms of this effect, we examined the impact of JTD on the expression of AGEs and RAGE in diabetic kidneys using an ELISA and Western blot. Figure 5e, f reveal that after 12 weeks of treatment, AGEs and RAGE in the serum were lower in the mice treated with JTD in a dose-dependent manner, compared to the model group. Furthermore, when detecting the expression of AGEs and RAGE in the kidney by Western blot, we found that JTD alleviated the accumulation of both (Fig. 5h, i).

### JTD down-regulates inflammatory factors

**Fig. 6:**
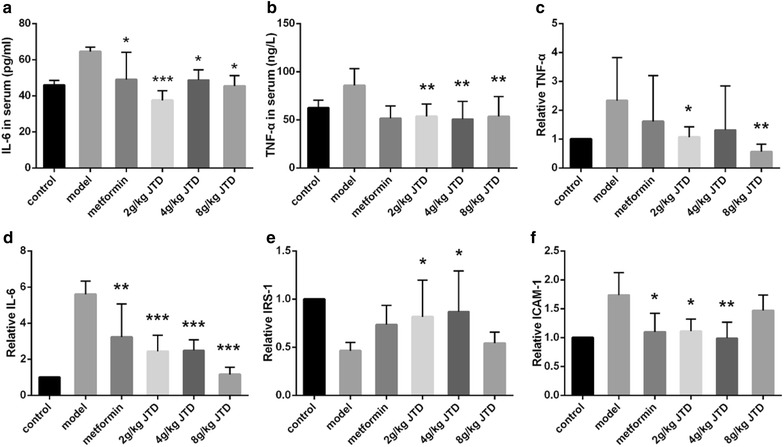
JTD down-regulates inflammatory factors. **a** JTD reduces the level of IL-6 in the serum; **b** JTD reduces the level of TNF-α in the serum; **c** JTD reduces the expression of TNF-α mRNA in the kidney; **d** JTD reduces the expression of IL-6 mRNA in the kidney; **e** JTD reduces the expression of IRS-1 mRNA in the kidney; **f** JTD reduces the expression of ICAM-1 mRNA in the kidney. Data are expressed as the mean ± S.D.; *N* = 10; *P < 0.05; **P < 0.01; and ***P < 0.001 compared to the model group

DN is confirmed to be an inflammatory disease, and pro-inflammatory cytokines are closely related to the pathology of DN. The increased production of free radicals and pro-inflammatory mediators (e.g., lipid mediators and cytokines) aggravates inflammation and damages host tissues. Thus, we detected the concentration of inflammatory factors to determine whether JTD treatment affects renal inflammation under diabetic conditions. The levels of IRS-1, TNF-α, IL-6, and ICAM-1 were detected with real-time PCR or an ELISA. As shown in Fig. 6e, IRS-1 expression was decreased in the model group, while the 12-week administration of metformin and JTD up-regulated IRS-1. Additionally, treatment with JTD down-regulated the expression of IL-6 in the serum (Fig. 6a), which was increased in the model group. Furthermore, treatment with JTD decreased the level of IL-6 mRNA in the kidney in a dose-dependent manner (Fig. 6d). TNF-α was highly expressed in the model group, while the administration of metformin and JTD was found to normalize both the mRNA and protein expression of TNF-α in the serum and kidney (Fig. 6b, c). In addition, ICAM-1 mRNA was found to be down-regulated by treatment with metformin and JTD (Fig. 6f).

### JTD activates the PI3K/Akt signaling pathway and inhibits NF-κB signaling pathways

**Fig. 7:**
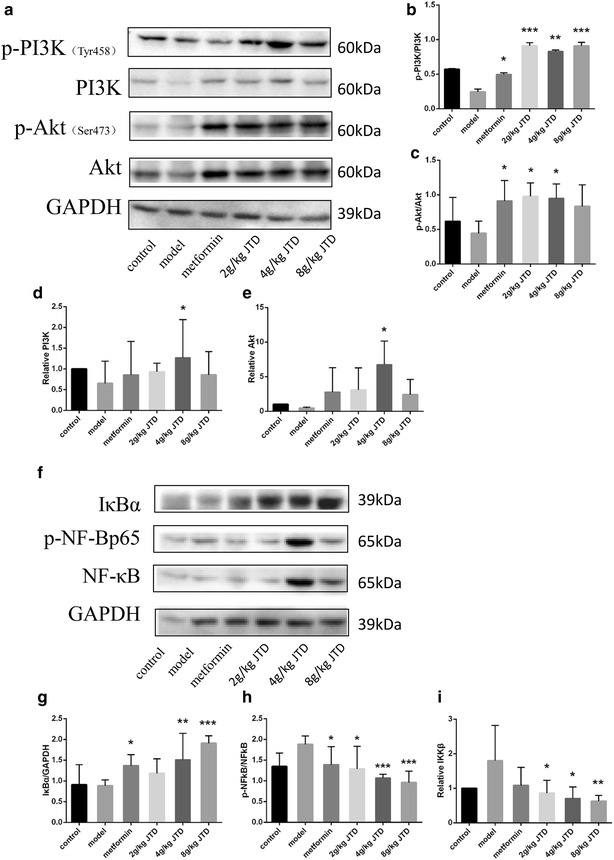
JTD activates the PI3K/Akt signaling pathway and inhibits NF-κB signaling pathways. **a** Representative Western blots of phosphorylated PI3K (Tyr 458), PI3K p85, phosphorylated Akt (Ser 473), Akt (pan), and GAPDH proteins isolated from the mouse kidneys of different groups. Protein bands shown are representative of >3 independent experiments with similar results. **b** JTD up-regulates the phosphorylation of PI3K; **c** JTD up-regulates the amount of phosphorylated Akt (Ser473); **d** JTD up-regulates PI3K p85mRNA expression; **e** JTD up-regulates the mRNA expression of Akt (pan); **f** representative Western blots of IκBα as well as phosphorylated NF-κB p65, NF-κB p65, and GAPDH proteins isolated from the mouse kidneys of different groups. The protein bands are representative of >3 independent experiments with similar results. **g** JTD down-regulates the protein expression of IκBα in the kidney; **h** JTD downregulates the phosphorylation of NF-κB p65; **i** JTD down-regulates the mRNA expression of IKKβ. Data are expressed as the mean ± S.D.; *N* = 10; *P < 0.05; **P < 0.01; and ***P < 0.001 compared to the model group

PI3K/Akt is closely related to the progression of DN, which is considered to be an inflammatory disease [4]. Moreover, NF-κB serves as a vital mediator of the inflammatory response. IKKβ and IκBα are two important modulators upstream of the NF-κB signal transduction cascade. IKKβ acts as a protein subunit of IκBα, and IκBα retains NF-κB in an inactive state in the cytoplasm. Thus, we detected the effect of JTD on the PI3K/Akt and NF-κB signaling pathways. The level of p-PI3K (Tyr 458), PI3K p65, p-Akt (Ser 473), Akt (pan), p-NF-κB p65, NF-κB p65, IκBα, and IKKβ were detected via Western blotting or real-time PCR. Figure 7d, e show that in mice treated with metformin and JTD group for 12 weeks, the level of PI3K and Akt were up-regulated, and there was a statistically significant difference between the group that received the medium dose of JTD and the model group. Furthermore, p-PI3K/PI3K and p-Akt/Akt were also up-regulated in the group treated with metformin and JTD (Fig. 7a–c). Treatment with JTD can up-regulate the expression of PI3K and Akt. Figure 7g showed that in the mice treated with JTD for 12 weeks, the level of IκBα was up-regulated, while the level of IKKβ mRNA and p-NF-κB/NF-κB protein are down-regulated (Fig. 7h, i).

## Discussion

DN is a leading cause of end-stage renal disease with limited effective therapies, and affects up to 40% of patients with type 1 or type 2 DM [15]. As DN is the strongest predictor of mortality in diabetic patients worldwide [15], it is critical to study the mechanism of DN in conjunction with strategies for prevention and treatment. Although knowledge regarding the molecular mechanisms of DN has progressed in recent years [16–18], efficient therapeutic approaches that prevent or reverse the progression of DN are lacking, highlighting the need to identify new treatment strategies.

**Fig. 8:**
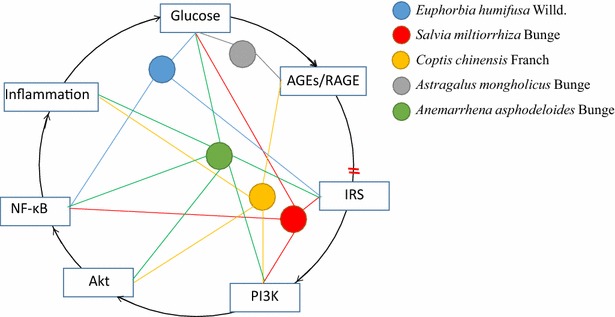
Mechanism of JTD components on DN

**Fig. 9:**
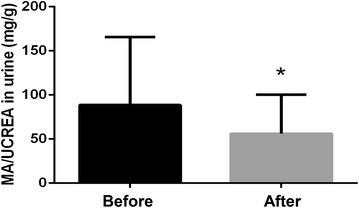
JTD reduces MA/UCREA in DN patients after 12 weeks administration. *P < 0.05; **P < 0.01; and ***P < 0.001 compared with the patients before treatment

In China, TCMs are known to produce remarkable results and some TCMs are recommended for the treatment of DN [19–21]. Unlike single compounds with only one active chemical ingredient, TCMs are typically complex combinations of chemicals that act in concert. Moreover, any one of the active components of a TCM alone cannot reflect the ultimate therapeutic effect of the entire medicine [22, 23]. Previous studies show that TCMs that exhibit heat- and toxin-clearing effects can down-regulate blood glucose, promote circulation, and relieve the clinical indicators of DN [20]. Several studies suggest that TCMs might exert hypoglycemic effects through anti-inflammatory mechanisms, including the modulation of inflammatory factors, AGEs, renin-angiotensin systems, and lipid metabolism [11]. In addition, JTD contains various plant components that are widely used in TCM, many of which are effective in reducing both glucose and inflammation (Fig. 8). *Euphorbia humifusa* Willd is one of the elements of JTD thought to reduce FG and enhance insulin sensitivity in KK-Ay mice [24]. Additionally, it can inhibit NF-κB activity and down-regulate the production of inflammatory mediators, such as nitric oxide (NO) and TNF-α [25, 26]. *Salvia miltiorrhiza* Bunge has been reported to improve the quantity and amount of pancreatic islet cells, repair their structure and function, increase glucose transporter 4 (GLUT4) and glycogen synthase protein expression in the skeletal muscle, as well as suppress the secretion of NO, TNF-α, and IL-6 in RAW264.7 macrophages stimulated with LPS [12]. Additionally, it has been reported that *S. miltiorrhiza* Bunge inhibits platelet activation and arterial thrombosis via regulation of the PI3K and NF-κB signaling pathways [24]. Raw *A. mongholicus* Bunge, which tonifies qi, was commonly used clinically to reduce diabetic symptoms, such as fatigue; it was also found to increase serum levels of total adiponectin, as well as alleviate hyperglycemia, glucose intolerance, and IR in obese mice [27]. It was also reported to attenuate DN by reducing endoplasmic reticulum stress [28]. Similarly, *A. asphodeloides* Bunge was reported to reduce FG and improve impaired glucose tolerance in type 2 DM rats, and the flavonoids of *A. asphodeloides* *Bunge* are thought to reduce glucose and TGs [13]. Furthermore, TB-II, a main ingredient of *A. asphodeloides* Bunge, notably ameliorated IR and inflammation, and significantly improved cell viability decreased TNF-α and IL-6 levels, and the expression of p-NF-κBp65, p-IKKβ, p-IRS-1, p-PI3K, and p-Akt [13]. *Coptis chinensis Franch* was confirmed to significantly inhibit all the three periods of nonenzymatic protein glycation in vitro, including amadori products, dicarbonyl compounds, and AGEs formation [29]. It is also reported to attenuate myocardial ischemia–reperfusion by regulating the PI3K/Akt signaling pathway and the subsequent reduction of inflammatory factors, including IL-6, IL-18, and TNF-α [30]. In our previous random control trials and animal experiments, we confirmed that JTD is safe and effective at reducing glucose and MA/UCREA in both patients and KK-Ay mice (accepted). In this study, we found that JTD can reduce the morphological changes in renal tissue, ameliorate glucose and lipid metabolism dysfunction, as well as decrease MA, UN, and Cr. Moreover, JTD can also decrease the enhanced levels of blood glucose and the accumulation AGEs and RAGE, thereby normalizing IR. In our study, the increased phosphorylation of PI3K-Akt and IκBα expression decreased IKKβ, and reduced phosphorylation of NF-κB was observed in the kidneys of JTD-treated KK-Ay mice. In addition, we found that alterations in a number of indexes of renal inflammation (e.g., increased ICAM-1, TNF-α, and IL-6 expression) in KK-Ay mice were alleviated by JTD treatment. These results suggest that the activation of PI3K/Akt, inhibition of the NF-κB signaling pathway, and NF-κB-dependent inflammation may be a key mechanism by which JTD attenuates DN (Fig. 9).

DM is considered to be a group of metabolic diseases characterized by a high concentration of blood glucose, and glucose glycates proteins that give rise to AGEs, which induce cellular effects through interacting with specific cellular receptors. RAGE is an important signal transduction receptor known to activate an array of signal transduction cascades in response to the binding of AGEs [31]. Moreover, AGEs and RAGE play critical roles in the pathogenesis of DN [31]. AGEs result in cellular oxidative stress and IR, which have been implicated as causative factors in diabetic complications [4]. The accumulation of AGEs and RAGE can induce harmful changes to some protein structures, thereby decreasing their enzymatic activity and interfering matrix protein connection, which can cause dysfunction in renal cells and induce histological changes, including inflammation, focal glomerulosclerosis, mesangial expansion, tubulointerstitial fibrosis, and epithelial- to- mesenchymal transdifferentiation [3]. It has been confirmed that elevated levels of AGEs and RAGE have been found to activate pro-inflammatory NF-κB, an initial step in type 2 DM evolution that contributes to both β-cell apoptosis and IR [32], and induces the release of inflammatory factors [33]. IRS-1 and PI3K are key insulin signaling molecules critically involved in glucose metabolism and IR [34, 35]. One of the key mechanisms observed in the tissues impacted by type 2 DM is that the phosphorylation of serine residues in the insulin receptor and IRS-1 molecule results in diminished enzymatic activity in the PI3K/Akt pathway [33]. When insulin binds to the insulin receptor (InsR), it stimulates InsR intrinsic kinase activity and subsequently activates PI3K and Akt in succession [33]. Therefore, due to an increase in insulin receptor activity, PI3K/Akt signaling is activated in the renal cortex of db/db mice during the early phase of DM [33]. Additionally, PI3K/Akt signaling was found to be lower in the glomeruli of 12-week-old db/db mice, which has been linked to the death of podocytes in db/db mice [36]. The down-regulation of PI3K/Akt signaling contributes to renal tubular apoptosis in the kidneys of STZ-induced diabetic mice and apoptosis in the proximal tubular cells of the kidney [37]. Mounting evidence has highlighted that DM activates NF-κB signaling, which regulates the expression of numerous genes that play key roles in the inflammatory response during kidney injury [5, 38, 39]. Moreover, the inhibition of NF-κB has been shown to result in the significant improvement of DN [40, 41]. Therefore, we postulated that the inactivation of NF-κB was involved in the JTD-mediated protection against DN. Under steady-state conditions, NF-κB is present in the cytoplasm and bound to its inhibitory protein, IκB; however, when IκB is phosphorylated by IKKβ, NF-κB is then released from the inhibitory unit, translocated into the nucleus, and undergoes phosphorylation. Finally, NF-κB promotes the transcription of pro-inflammatory cytokines, such as TNF-α, IL-6, and ICAM-1 [42]. A growing number of studies have revealed that the serum and urinary concentrations of TNF-α in patients with DN are substantially higher than that found in non-diabetic individuals, and TNF-α levels are implicated in the process of renal hypertrophy and hyperfunction during the initial stages of DN [43]. IL-6 is confirmed to be increased in patients with DN, and its levels are higher in patients with overt proteinuria compared to those exhibiting microalbuminuria or normoalbuminuria [44]. Additionally, recent work also found that increased IL-6 is associated with glomerular basement membrane (GBM) thickening and mesangial expansion in the kidney biopsies of DM patients [45]. Produced by renal mesangial cells, IL-6 is thought to mediate endothelial permeability and mesangial proliferation [46]. Additionally, an ICAM-1 deficiency in transgenic mice results in a substantial decrease in macrophage accumulation in the glomeruli and a reduction in glomerular hypertrophy [42].

In our study, we found that the concentration of AGEs and RAGE in a KK-Ay mouse model were higher than that of the normal controls. In addition, the expression of IRS-1, PI3K/Akt, and NF-κB, as well as the histological changes observed in the KK-Ay model group, are consistent with previous reports [3, 24, 36]. The findings of the present study suggest that JTD can relieve DN by down-regulating increased levels of blood glucose, so as to reduce the accumulation of AGEs and RAGE, as well as alleviate inflammation via activation of the PI3K/Akt signaling pathways and inhibiting NF-κB signaling. Additionally, this study demonstrates that PI3K/Akt-mediated NF-κB signaling might be a mechanism for the treatment of DN, and use of multiple herbal compounds may be a useful therapeutic approach. While unlike single compounds with only one active chemical ingredient, Traditional Chinese Medicine, especially compounds are typically complicated combinations of chemicals that act in concert. The ultimate therapeutic effect of the compound is multi-targets and the effect might vary as the concentration change. Thus the effect of compound might not all be in a dose dependent manner. However, since the evidence demonstrating the effect of JTD on DN was obtained from studies using animal models, these findings should be confirmed with further research in diabetic patients.

## Conclusion

JTD is a promising agent for the treatment of DN, and its therapeutic mechanism is likely related to the regulation of AGEs, RAGE, and PI3K/Akt-mediated NF-κB signaling pathways.

## Additional file

**Additional file 1.** The minimum standards of reporting checklist.

## Abbreviations

JTD: jiangtang decoction
DM: diabetes mellitus
DN: diabetic nephropathy
TGs: triglycerides
TC: total cholesterol
FG: fasting glucose
PPG: postprandial glucose
OGTT: oral glucose tolerance test
ITT: insulin tolerance test
AUC: area under the curve
MA: microalbumin
MA/UCREA: microalbumin/urine creatinine
UN: urea nitrogen
Cr: creatinine
IL-6: interleukin-6
TNF-α: tumor necrosis factor-alpha
ICAM-1: intercellular adhesion molecule-1
p-PI3K: phosphorylated phosphatidylinositol 3-kinase
PI3K: phosphatidylinositol 3-kinase
p-Akt: phosphorylated protein kinase B
Akt: protein kinase B
IKKβ: IκB kinase-beta antibody
IκBα: nuclear factor of kappa light polypeptide gene enhancer in B-cells inhibitor, alpha
p-NF-κB: phosphorylated nuclear factor kappa-B
NF-κB: nuclear factor kappa-B
AGEs: advanced glycation end products
RAGE: receptor of glycation end products
H&E: hematoxylin-eosin staining
PASM: periodic schiff-methenamine
EM: electronic microscopy
GLUT4: glucose transporter 4
NO: nitric oxide
GBM: glomerular basement membrane

## References

[1] Dronavalli S, Duka I, Bakris GL (2008). The pathogenesis of diabetic nephropathy. Nat Clin Pract J Clin Endocr Metab.

[2] Ramasamy R, Yan SF, Herold K, Clynes R, Schmidt AM (2008). Receptor for advanced glycation end products: fundamental roles in the inflammatory response: winding the way to the pathogenesis of endothelial dysfunction and atherosclerosis. Ann N Y Acad Sci.

[3] Han C, Lu Y, Wei Y, Wu B, Liu Y, He R (2014). D-ribosylation induces cognitive impairment through RAGE-dependent astrocytic inflammation. Cell Death Dis.

[4] Tuttle KR (2005). Linking metabolism and immunology: diabetic nephropathy is an inflammatory disease. J Am Soc Nephrol.

[5] Navarro-González JF, Mora-Fernández C, De Fuentes MM, García-Pérez J (2011). Inflammatory molecules and pathways in the pathogenesis of diabetic nephropathy. Nat Rev Nephrol.

[6] Cantley LC (2002). The phosphoinositide 3-kinase pathway. Science.

[7] Zhu J, Sun N, Aoudjit L, Li H, Kawachi H, Lemay S, Takano T (2008). Nephrin mediates actin reorganization via phosphoinositide 3-kinase in podocytes. Kidney Int.

[8] Yu S, Li Y (2013). Dexamethasone inhibits podocyte apoptosis by stabilizing the PI3K/Akt signal pathway. Biomed Res Int.

[9] Zhu J, Wu Y, Wang Y (2012). Multivariate analysis of Chinese medicine syndrome type and its correlated factors of diabetic nephropathy. Mod J Integr Tradit Chin West Med.

[10] Hu J, Pang W (2013). Hypoglycemic effect of polysaccharides with different molecular weight of *Pseudostellaria heterophylla*. Complement Altern Med.

[11] Xie W, Du L (2011). Diabetes is an inflammatory disease: evidence from traditional Chinese medicines. Diabetes Obes Metab.

[12] Huang MQ, Zhou CJ, Zhang YP (2016). Salvianolic acid B ameliorates hyperglycemia and dyslipidemia in db/db mice through the AMPK pathway. Cell Physiol Biochem.

[13] Yuan YL, Lin BQ, Zhang CF (2016). Timosaponin B-II ameliorates palmitate-induced insulin resistance and inflammation via IRS-1/PI3K/Akt and IKK/NF-κB pathways. Am J Chin Med.

[14] Pugliese G, Pricci F, Romeo G, Pugliese F, Mene P, Giannini S (1997). Upregulation of mesangial growth factor and extracellular matrix synthesis by advanced glycation end products via a receptor-mediated mechanism. Diabetes.

[15] Martinez-Castelao A, Navarro-Gonzalez JF, Gorriz JL, de Alvaro F (2015). The concept and the epidemiology of diabetic nephropathy have changed in recent years. J Clin Med.

[16] Reidy K, Kang HM, Hostetter T, Susztak K (2014). Molecular mechanisms of diabetic kidney disease. J Clin Investig.

[17] Zoja C, Zanchi C, Benigni A (2015). Key pathways in renal disease progression of experimental diabetes. Nephrol Dial Transplant.

[18] Forbes JM, Cooper ME (2013). Mechanisms of diabetic complications. Physiol Rev.

[19] The Microvascular Complications Group of Diabetes Association of the Chinese Medical Association (2014). The expert consensus of diabetic kidney disease prevention and control. Chin J Diabetes Mellit.

[20] Sun GD, Li CY, Cui WP, Guo QY, Dong CQ, Zou HB (2016). Review of herbal traditional chinese medicine for the treatment of diabetic nephropathy. J Diabetes Res..

[21] Liu X, Liu L, Chen P, Zhou L, Zhang Y, Wu Y (2014). Clinical trials of traditional Chinese medicine in the treatment of diabetic nephropathy—a systematic review based on a subgroup analysis. J Ethnopharmacol.

[22] Gao H, Sun W, Zhao J (2016). Tanshinones and diethyl blechnics with anti-inflammatory and anti-cancer activities from *Salvia miltiorrhiza* Bunge (Danshen). Sci Rep.

[23] Hu L, Luan LJ, Cheng YY (2004). Study on establishing the correlativity between fingerprint peaks and the effective fraction of traditional Chinese medicine prescription and its relevant herbs. Chin Pharm J.

[24] Wang LL, Fu H, Li WW, Song FJ, Song YX, Yu Q (2015). Study of the effect of Euphorbia Herb on alleviating insulin resistance in type 2 diabetic model KK-Ay mice. China J Chin Materia Med.

[25] Shin SY, Kim CG, Jung YJ (2016). *Euphorbia humifusa* Willd exerts inhibition of breast cancer cell invasion and metastasis through inhibition of TNFα-induced MMP-9 expression. BMC Complement Altern Med.

[26] Luyen BT, Tai BH, Thao NP (2014). Anti-inflammatory components of *Euphorbia humifusa* Willd. Bioorg Med Chem Lett.

[27] Xu A, Wang H, Hoo RL (2009). Selective elevation of adiponectin production by the natural compounds derived from a medicinal herb alleviates insulin resistance and glucose intolerance in obese mice. Endocrinology.

[28] Wang ZS, Xiong F, Xie XH (2015). Astragaloside IV attenuates proteinuria in streptozotocin-induced diabetic nephropathy via the inhibition of endoplasmic reticulum stress. BMC Nephrol.

[29] Yang Y, Li Y, Yin D (2016). *Coptis chinensis* polysaccharides inhibit advanced glycation end product formation. J Med Food.

[30] Qin-Wei Z, Yong-Guang LI (2016). Berberine attenuates myocardial ischemia reperfusion injury by suppressing the activation of PI3K/AKT signaling. Exp Ther Med.

[31] Wei Y, Han CS, Zhou J, Liu Y, Chen L, He RQ (2012). D-ribose in glycation and protein aggregation. Biochim Biophys Acta.

[32] Mark AB, Poulsen MW, Andersen S (2014). Consumption of a diet low in advanced glycation end products for 4 weeks improves insulin sensitivity in overweight women. Diabetes Care.

[33] Navarro-González JF, Mora-Fernández C (2008). The role of inflammatory cytokines in diabetic nephropathy. J Am Soc Nephrol.

[34] Zhu Y, Pereira RO, O’Neill BT (2012). Cardiac PI3K-Akt impairs insulin-stimulated glucose uptake independent of mTORC1 and GLUT4 translocation. Mol Endocrinol.

[35] Fukushima T, Arai T, Ariga-Nedachi M (2011). Insulin receptor substrates form high-molecular-mass complexes that modulate their availability to insulin/insulin-like growth factor-I receptor tyrosine kinases. Biochem Biophys Res Commun.

[36] Feliers D, Duraisamy S, Faulkner JL, Duch J, Lee AV, Abboud HE, Choudhury GG, Kasinath BS (2001). Activation of renal signaling pathways in db/db mice with type 2 diabetes. Kidney Int.

[37] Tejada T, Catanuto P, Ijaz A, Santos JV, Xia X, Sanchez P, Sanabria N, Lenz O, Elliot SJ, Fornoni A (2008). Failure to phosphorylate AKT in podocytes from mice with early diabetic nephropathy promotes cell death. Kidney Int.

[38] Sanz AB, Sanchez-Niño MD, Ramos AM, Moreno JA, Santamaria B, Ruiz-Ortega M, Egido J, Ortiz A (2010). NF-κB in renal inflammation. J Am Soc Nephrol.

[39] Rane MJ, Song Y, Jin S, Barati MT, Wu R, Kausar H, Tan Y, Wang Y, Zhou G, Klein JB (2010). Interplay between Akt and p38 MAPK pathways in the regulation of renal tubular cell apoptosis associated with diabetic nephropathy. Am J Physiol Renal Physiol.

[40] Ohga S, Shikata K, Yozai K (2007). Thiazolidinedione ameliorates renal injury in experimental diabetic rats through anti-inflammatory effects mediated by inhibition of NF-κB activation. Am J Physiol Renal Physiol.

[41] Lee FT, Cao Z, Long DM (2004). Interactions between angiotensin II and NF-κB-dependent pathways in modulating macrophage infiltration in experimental diabetic nephropathy. J Am Soc Nephrol.

[42] Chow FY, Nikolic-Paterson DJ, Ozols E, Atkins RC, Tesch GH (2005). Intercellular adhesion molecule-1 deficiency is protective against nephropathy in type 2 diabetic db/db mice. J Am Soc Nephrol.

[43] Navarro JF, Mora-Fernández C (2006). The role of TNF-α in diabetic nephropathy: pathogenic and therapeutic implications. Cytokine Growth Factor Rev.

[44] Aso Y, Yoshida N, Okumura K (2004). Coagulation and inflammation in overt diabetic nephropathy: association with hyperhomocysteinemia. Clin Chim Acta.

[45] Dalla M, Mussap M, Gallina P (2005). Acute-phase markers of inflammation and glomerular structure in patients with type 2 diabetes. J Am Soc Nephrol.

[46] Lim AK, Tesch GH (2012). Inflammation in diabetic nephropathy. Mediat Inflamm.

